# Gregarines impact consumption and development but not glucosinolate metabolism in the mustard leaf beetle

**DOI:** 10.3389/fphys.2024.1394576

**Published:** 2024-05-01

**Authors:** Alessa Barber, Jeanne Friedrichs, Caroline Müller

**Affiliations:** ^1^ Department of Chemical Ecology, Bielefeld University, Bielefeld, Germany; ^2^ Joint Institute for Individualisation in a Changing Environment (JICE), University of Münster and Bielefeld University, Bielefeld, Germany

**Keywords:** Chrysomelidae, detoxification, glucosinolates, gregarine infection, food consumption, development

## Abstract

Gregarines are usually classified as parasites, but recent studies suggest that they should be viewed on a parasitism-mutualism spectrum and may even be seen as part of the gut microbiota of host insects. As such, they may also impact the consumption of their hosts and/or be involved in the digestion or detoxification of the host’s diet. To study such effects of a gregarine species on those traits in its host, the mustard leaf beetle (*Phaedon cochleariae*) was used. This beetle species feeds on Brassicaceae plants that contain glucosinolates, which form toxic compounds when hydrolyzed by myrosinases. We cleaned host eggs from gametocysts and spores and reinfected half of the larvae with gregarines, to obtain gregarine-free (G-) and gregarine-infected (G+) larvae. Growth and food consumption parameters of these larvae were assessed by rearing individuals on watercress (*Nasturtium officinale*, Brassicaceae). A potential involvement of gregarines in the glucosinolate metabolism of *P. cochleariae* larvae was investigated by offering G- and G+ larvae leaf discs of watercress (containing mainly the benzenic 2-phenylethyl glucosinolate and myrosinases) or pea (*Pisum sativum*, Fabaceae, lacking glucosinolates and myrosinases) treated with the aliphatic 4-pentenyl glucosinolate or the indole 1-methoxy-3-indolylmethyl glucosinolate. Larval and fecal samples were analyzed via UHPLC-QTOF-MS/MS to search for breakdown metabolites. Larval development, body mass, growth rate and efficiency to convert food into body mass were negatively affected by gregarine infection while the pupal mass remained unaffected. The breakdown metabolites of benzenic and aliphatic glucosinolates were conjugated with aspartic acid, while those of the indole glucosinolate were conjugated with glutamic acid. Gregarine infection did not alter the larvae’s ability to metabolize glucosinolates and was independent of plant myrosinases. In summary, some negative effects of gregarines on host performance could be shown, indicating parasitism. Future studies may further disentangle this gregarine-host relationship and investigate the microbiome potentially involved in the glucosinolate metabolism.

## 1 Introduction

Parasitism describes the relationship between two species, in which one partner is negatively affected while the other gains a benefit. It is of widespread occurrence and displayed in numerous taxa (Poulin, 2011). One phylum commonly associated with parasitism is the unicellular Apicomplexa. In humans, for example, the apicomplexans *Plasmodium* spp. and *Toxoplasma gondii* cause malaria and toxoplasmosis, respectively (Schrével and Desportes, 2016; Boisard and Florent, 2020). Non-vertebrates are often parasitized by gregarines that also belong to the Apicomplexa (Schrével and Desportes, 2016; Rueckert et al., 2019; Boisard and Florent, 2020). Gregarines display a high host specificity (Rueckert et al., 2018), thus providing potential for coevolution between the gregarine and its host species (Schrével and Desportes, 2016). This coevolution sparks the discussion of considering gregarines as part of the gut microbiota (Rueckert et al., 2019). This notion is supported by reports of positive effects of gregarines on their hosts, contradicting the more common classification as parasites (Hecker et al., 2002; Arcila and Meunier, 2020). The effects of gregarines should therefore rather be viewed on a spectrum ranging from parasitism to mutualism (Rueckert et al., 2019). Moreover, their effects may depend on their impact on the host metabolism as well as on the presence of other stresses influencing the physiological constitution of the host (Rodriguez et al., 2007; Wolz et al., 2022a). For example, the dragonfly *Libellula pulchella* (Odonata: Anisoptera) showed abnormalities in lipid and carbohydrate metabolism and was partly insulin-resistant when infected with gregarines (Schilder and Marden, 2006). In the caeca of *Trichosia pubescens* (Diptera: Sciaridae), proteins increased in cells infected with gregarines, suggesting an adaption of the host cell metabolism to maintain the growth of the symbiont (Malavasi et al., 1976). However, little is known to what extend gregarines influence the consumption by their host and what role they play in the host’s digestion or detoxification of the diet.

For many herbivores, plant material only becomes accessible after microbial biotransformation (Dearing et al., 2022). Plant toxins can pose an even greater challenge when it comes to acquiring nutrients (Dearing et al., 2022). Here, the gut microbiome may be involved in the defense against toxic specialized metabolites (Hammer and Bowers, 2015), as has been demonstrated for isothiocyanates (ITCs), caffeine and flavonoids (Dearing et al., 2022). For example, antibiotic-fed individuals of *Psylliodes chrysocephala* (Coleoptera: Chrysomelidae) had a higher amount of unmetabolized ITCs in their feces than control beetles, suggesting a bacterial metabolism of ITCs (Shukla and Beran, 2020). Likewise, in feces of *Hylobius abietis* (Coleoptera: Curculionidae) treated with antibiotics, more diterpenes were found than in untreated individuals (Berasategui et al., 2017). After reinfection with the gut bacteria of these antibiotic-treated beetle species, the capacity to metabolize isothiocyanates and diterpenes was restored (Berasategui et al., 2017; Shukla and Beran, 2020). In contrast, the larval metabolism of *Melitaea cinxia* (Lepidoptera: Nymphalidae) was not affected by a treatment with antibiotics (Duplouy et al., 2020). To what extend gregarines may be involved in countering harmful phytochemicals is poorly understood.

Hosts usually infect themselves with gregarines through ingestion of spores that have been excreted with the feces of infected conspecifics (Gigliolli et al., 2016). The sporozoites develop into trophozoites, which attach to the gut wall (Kim et al., 2014; Gigliolli et al., 2016). The gregarine trophozoites take up nutrients from their hosts (Valigurová and Florent, 2021), which can have effects on the growth and development of the hosts. For example, larvae of *Tribolium castaneum* (Coleoptera: Tenebrionidae) infected with gregarines were smaller than uninfected ones and did not reach pupation, possibly due to an occlusion of the midgut by the gregarines (Gigliolli et al., 2016). In the damselfly *Calopteryx splendens xanthostoma* (Zygoptera: Calopterygidae), males with a high gregarine load showed lower fat contents than those with a low gregarine load (Siva-Jothy and Plaistow, 1999). The leaf beetle *Phaedon cochleariae* (Coleoptera: Chysomelidae) can be infected by the gregarine species *Gregarina cochlearium* (Wolz et al., 2022a), which has negative, neutral or positive effects on its host under different circumstances. In combination with food deprivation or a sublethal insecticide exposure, gregarine–infected individuals of *P. cochleariae* had a lower survival than non-infected individuals, indicating a negative effect of gregarines on their host (Wolz et al., 2022a; Wolz et al., 2022b). Under benign conditions, developmental time from larvae to adult beetles was either prolonged (Wolz et al., 2022b) or remained unaffected (Wolz et al., 2022a). Reproduction and adult body mass were unaffected by the gregarine infection status of *P. cochleariae* when not exposed to another stress, while the survival probability was even enhanced in infected individuals (Wolz et al., 2022b), indicating potential positive effects.

Larvae and adults of *P. cochleariae* feed on Brassicaceae plants, which are known for their glucosinolate-myrosinase defense system. Glucosinolates are specialized metabolites that originate from different precursor amino acids, resulting in benzenic, aliphatic or indole glucosinolates (Blažević et al., 2020). They are hydrolyzed into ITCs, nitriles (CNs) or other products by myrosinases (Blažević et al., 2020). These hydrolysis products have repellent and toxic effects on most herbivore species (Fahey et al., 2001; Nguyen et al., 2020). Some herbivore species specialized on Brassicaceae circumvent the formation of toxic compounds by, for example, converting glucosinolates to desulfoglucosinolates (Ratzka et al., 2002; Manivannan et al., 2021), using CN-specifier proteins to convert glucosinolates into CNs which can then be excreted (Wittstock et al., 2004) or sequestration of glucosinolates into other body parts such as the hemolymph (Abdalsamee et al., 2014). Many more different strategies of glucosinolate metabolism are known for different insects (Friedrichs et al., 2022). Larvae and adults of *P. cochleariae* conjugate intermediate breakdown products of benzenic and indole glucosinolates, i.e., ITCs and CNs usually formed by myrosinases, with aspartic acid and glutamic acid, respectively (Friedrichs et al., 2020; Friedrichs et al., 2022). Interestingly, the same conjugation products are formed when plant myrosinases are absent, indicating that the first hydrolysis step to ITC or CN before the conjugation can happen in the beetles (Friedrichs et al., 2020; Friedrichs et al., 2022). The question remains if gregarines may be involved in this metabolism.

In the present study we investigated whether gregarines have an effect on the larval food intake, conversion of food into body mass, larval development and glucosinolate metabolism by *P. cochleariae*. We expected that gregarine-infected larvae need to consume more food during their larval development to compensate for potential losses of nutrients caused by the gregarine infection, but still gain less body mass than uninfected conspecifics. An infection with gregarines may also cause a prolonged developmental time. We tested effects of a gregarine-infection on glucosinolate metabolism by the larvae using feeding experiments with different glucosinolates, i.e., the aliphatic 4-pentenyl glucosinolate (glucobrassicanapin) or the indole 1-methoxyindol-3-ylmethyl glucosinolate (neoglucobrassicin), applied on myrosinase-containing watercress and myrosinase-free pea leaves. We hypothesized that the larvae will metabolize the aliphatic glucosinolate in yet another way than in those already known for benzenic and indole glucosinolates (Friedrichs et al., 2022), as the side chains of glucosinolates influence their biological properties (Blažević et al., 2020). If gregarines are involved in the glucosinolate metabolism by *P. cochleariae*, we expected that the amino acid conjugates would not be formed by gregarine-free larvae feeding on myrosinase-free leaves.

## 2 Materials and methods

### 2.1 Plant and beetle rearing

Watercress (*Nasturtium officinale*, Brassicaceae) and pea plants (*Pisum sativum*, Fabaceae) were grown from seeds (watercress: Volmary GmbH, Münster, Germany; pea: “Kleine Rheinländerin” from Kiepenkerl, Bruno Nebelung GmbH, Konken, Germany) in a greenhouse (60% r.h., 16:8 h light:dark) in pots (12 cm diameter) on composted soil. For rearing and all experiments, 7–8 weeks old flowering watercress plants were used. Six-weeks-old non-flowering pea plants were used for the feeding assay investigating the glucosinolate metabolism. Beetles were reared for several generations in climate cabinets (20°C, 65% r.h., 16:8 h light:dark) in plastic rearing boxes (20 × 20 × 6.5 cm) with about 100–200 individuals per box. The gene pool was refreshed almost every year by introducing individuals that were captured in the wild (51° 51′ 21″ N, 8° 41’ 37″ E). Every other day beetles received fresh stems with leaves of watercress.

### 2.2 Infection with gregarines

The beetles in our laboratory stock are usually infected with the gregarine species *G. cochlearium* and we also found high natural infection levels in the field, but also some individuals without a gregarine infection (Barber, pers. communication). To obtain gregarine-free individuals, gametocysts and infectious spores were removed from the eggs of *P. cochleariae*, following Wolz et al. (2022a), (2022b). Eggs laid within a period of 24 h into the veins of watercress leaves (Müller and Rosenberger, 2006) were carefully removed from the leaves and cleaned on a filter paper with a brush and tap water. These eggs were randomly distributed among two rearing boxes. One of the boxes was assigned to the gregarine-free treatment (G-), the other to the gregarine-infected treatment (G+). One day before larval hatching (about 7 days after eggs were laid), stems of watercress already partly consumed for 24 h by infected beetles from the rearing stock were added to the G+ box to enable reinfection with gregarines. The hatching larvae were assumed to take up gregarine spores that had been excreted by conspecifics on the plant material. For the control group (G-), intact watercress stems were damaged slightly at the stem and the leaves with scissors and left in a box without any insects for 24 h to cause a comparable damage, before adding them to the G-box. Watercress stems of the respective treatments were added to the boxes for the first 2 days after larval hatching. From the third day onwards, both the G- and the G+ larvae received fresh, undamaged watercress leaf discs or stems every other day. One subset of larvae was used for the food consumption assay, another subset was reared in the boxes until molt to the second larval instar and then used for the feeding assay to investigate the glucosinolate metabolism. Remaining larvae of both treatments were reared until the third instar to determine their gregarine infection status. Therefore, *n* = 10 larvae of each gregarine treatment (and each box used) were frozen at −20°C at day 13 after hatching, following Wolz et al. (2022a). The larvae were dissected in sodium phosphate buffer (0.1 M; pH = 7.2) and their head and gut carefully removed with tweezers. The gut was opened with tweezers to make the trophozoits visible. All larvae in the G-treatment were uninfected, while all larvae of the G+ treatment were infected, with on average 527 ± 374 (mean ± SD, *n* = 50) trophozoits (counted in larvae used in the glucosinolate metabolism assay).

### 2.3 Measurements of larval food consumption and development in dependence of gregarine infection

To investigate the effects of gregarine infection on food consumption and larval development, G- and G+ larvae (*n* = 20 per treatment) were weighed 3 days after hatching (Sartorius microbalance ME36S, Sartorius AG Goettingen, Deutschland, 0.01 mg) and placed into separate Petri dishes (5.5 cm in diameter), lined with moistened filter paper. They were offered watercress leaf discs (2 cm in diameter), allowing for feeding *ad libitum*. A subset of leaf discs were weighed (*n* = 20) to obtain the mean mass per area. Every other day, leaf discs were exchanged with fresh ones and the filter paper was renewed and moistened. The leaf remains were scanned using *ImageJ* (https://imagej.nih.gov/ij/). The consumed area was calculated from the difference to the area of a complete leaf disc and converted into the leaf mass using the mean mass per area. The days until pupation were recorded by checking the state of the individuals once a day and pupae were weighed at that day. The efficiency of conversion of ingested food index [ECI (Eq. 1)] and growth rate [GR (Eq. 2)] were calculated using the following formulas (Waldbauer et al., 1984).

$$\text{ECI}=\frac{change\;in\;larval\;body\;mass\;\left(g\right)}{feeding\;amount\;\left(g\right)}*100\%$$ (1)

$$\text{GR}=\frac{change\;in\;larval\;body\;mass\;\left(g\right)}{mean\;body\;mass\;\left(g\right)*time\;\left(d\right)}$$ (2)

The feeding amount was the total amount of consumed leaf mass during the experimental time. Change in larval body mass was calculated as the difference between the initial larval body mass and the pupal body mass. The mean body mass was the mean of the initial larval body mass and the pupal body mass. Body mass and feeding amount were taken and calculated as fresh weights.

### 2.4 Feeding assays for investigation of glucosinolate metabolism in dependence of host plant species and gregarine infection

To investigate the metabolism of glucosinolates in dependence of host plant species, i.e., containing (watercress) or lacking (pea) internal myrosinases, and gregarine infection status, feeding experiments were carried out following Friedrichs et al. (2020), (2022). Pea is not a natural host plant of *P. cochleariae*, but the larvae feed on pea to some extent under laboratory conditions (Reifenrath and Müller, 2008), offering thus a suitable substrate to test glucosinolate metabolism independent of plant myrosinases. Larvae were taken directly after the molt to the second instar (4–5 days after hatching) to ensure that no myrosinase residues from watercress were left in the gut (Friedrichs et al., 2020) and were kept in groups of three of the same gregarine treatment in Petri dishes (5.5 cm in diameter, to allow larvae to quickly find the leaf disc and start feeding) lined with moistened filter paper. Larvae were starved for at least two and a maximum of 5 h.

**FIGURE 1:**
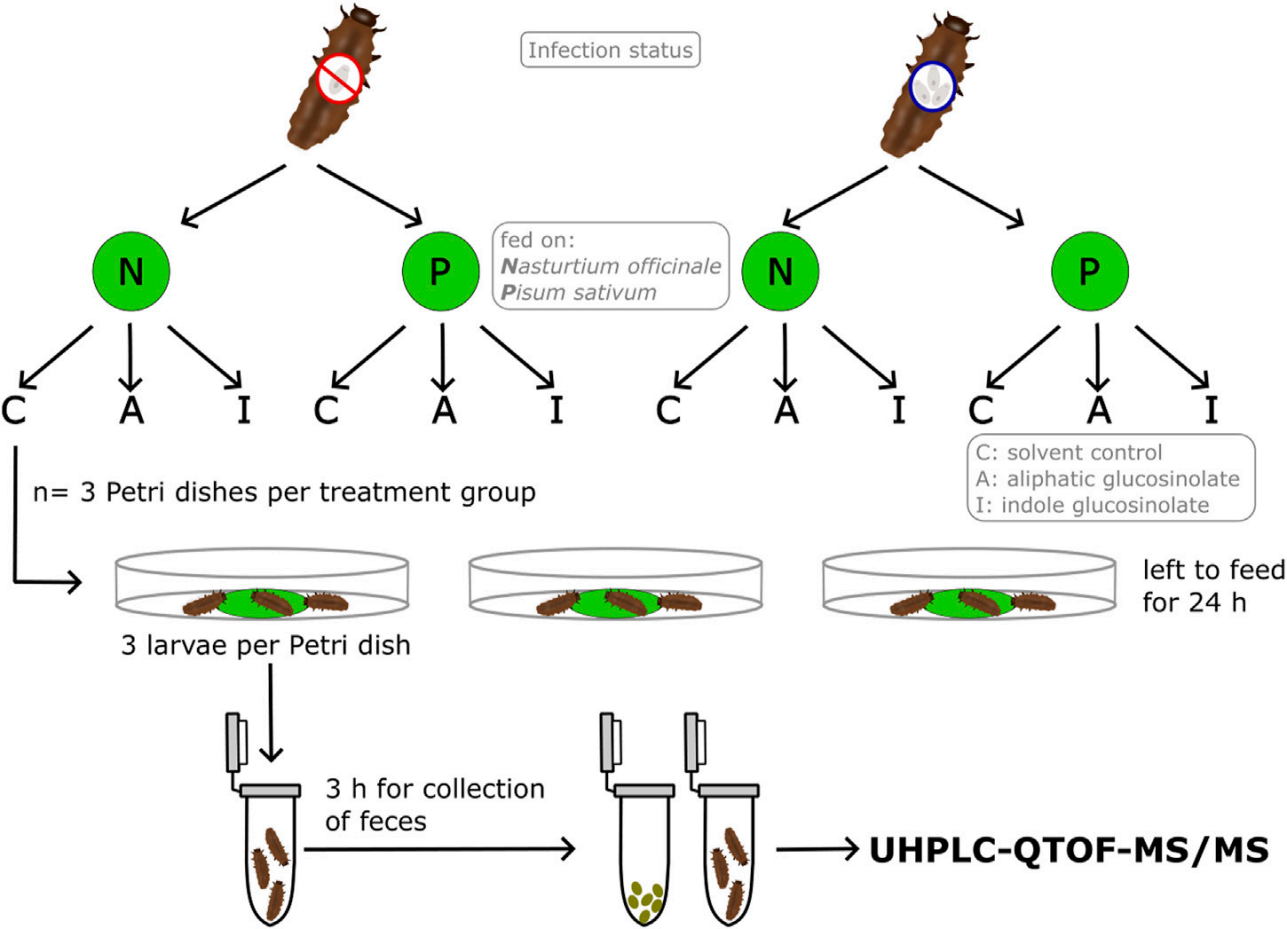
Illustration of the experimental set-up and the treatments for the glucosinolate metabolism experiment. Groups of three larvae of *Phaedon cochleariae* were either not infected or infected with gregarines and offered one leaf disc of watercress (*Nasturtium officinale*, N) or pea (*Pisum sativum*, P) treated with either only solvent as control (C), 4-pentenyl glucosinolate (A) or 1-methoxyindol-3-ylmethyl glucosinolate (I). Larvae were allowed to feed for 24 h or until ≥10% of the leaf disc were consumed and then transferred to an Eppendorf tube for collection of the feces for 3 h. Fecal and larval samples were then used for measurements with UHPLC-QTOF-MS/MS.

Leaf discs of watercress and pea (2 cm in diamter) were treated with 50 µL of a 20 mM solution of either the aliphatic 4-pentenyl glucosinolate or the indole 1-methoxyindol-3-ylmethyl glucosinolate (both Phytoplan, Heidelberg, Germany) diluted in a mixture of methanol (Fisher Scientific, Loughborough, United Kingdom): deionized water: dichlormethane (Merck KGaA, Darmstadt, Germany) in a relation of 64:30:6 (*v*:*v*:*v*). Thus, each leaf disc received 0.1 µmol of (additional) glucosinolate, which corresponds to amounts that can be found in leaves of Chinese cabbage (*Brassica rapa* L. ssp *pekinensis*) (Lee et al., 2014; Baek et al., 2016; Chun et al., 2018), a potential host plant for *P. cochleariae*. Previous feeding tests with the aliphatic 2-propenyl glucosinolate had not revealed any breakdown metabolites (Friedrichs et al., 2022). The ITC of 2-propenyl glucosinolate is particularly unstable (Ohta et al., 1995; Luang-In and Rossiter, 2015), while the ITC resulting from 4-pentenyl glucosinolate used in the present study may be somewhat more stable. Control leaves were treated with 50 µL of solvent mix only (in the following called “solvent control”). Solvents were allowed to evaporate for 40 min before adding one leaf disc to every Petri dish (*n* = 3 replicates per insect gregarine treatment, plant species and leaf treatment). The different treatment groups are depicted in Figure 1. Petri dishes were illuminated with light from above. Additional leaf discs were treated in the same way but were kept in a Petri dish (9 cm in diameter, to prevent leaves of the same plant species and solvent treatment from touching each other) without larvae to serve as a control (*n* = 3 per plant species and leaf treatment, called “control leaf”). Larvae were allowed to feed on the leaf discs for 24 h or until ≥10% of the leaf disc had been consumed in case of feeding tests with the non-host plant pea from which larvae fed much less. The consumed leaf area was calculated using millimeter paper. Control leaf discs without feeding of larvae were frozen in liquid nitrogen after 24 h. The groups of three larvae from each Petri dish were transferred into Eppendorf tubes to collect their feces. After 3 h, the larvae were transferred into new Eppendorf tubes. Both Eppendorf tubes with the feces and the larvae were then also frozen in liquid nitrogen. All samples were stored at −80°C until further processing.

### 2.5 Identification of metabolites

Samples of larvae, feces and control leaf discs were freeze-dried. Analyses of the glucosinolate metabolites were slightly modified from Friedrichs et al. (2020). To each sample, 300 µL of 90% methanol in deionized water containing 10 mg/L hydrocortisone (HPLC-Grade, Sigma-Aldrich Chemie GmbH, Steinheim, Germany) as internal standard was added. Samples were then homogenized using a pistil, vortexed manually for 5 s and centrifuged at 4°C for 5 min at 13,000 rpm. After that, samples were filtered (0.2 µm polytetrafluoroethylene membrane, 4 mm syringe filters, Phenomenex, Aschaffenburg, Germany) and transferred into vials. Samples were stored at −80°C until further analysis.

Potential glucosinolate metabolites were analyzed via ultra-high performance liquid chromatography with a diode array detector coupled to a quadrupole time of flight mass spectrometer (UHPLC-QTOF-MS/MS; UHPLC: Dionex UltiMate 3000, ThermoFisher Scientific, San José, CA, USA; QTOF: compact, Bruker Daltonics, Bremen, Germany). A Kinetex XB-C18 column (1.7 μm, 150 × 2.1 mm, Phenomenex, Torrance, CA, USA) with a guard column was used. Samples were separated by a gradient of eluent A: water with 0.1% formic acid (Carlo Erba reagents S.A.S., France, Val de Reuil) to eluent B: acetonitrile (LC-MS grade, Fisher Scientific, Loughborough, United Kingdom) with 0.1% formic acid at a flow rate of 0.5 mL min^-1^. The gradient started at 2% B and increased to 30% B in 20 min, then to 75% B in 9 min, followed by column cleaning and equilibration. The injection volume was 6 μL at an autosampler temperature of 5°C. The column oven was heated to 45°C. Line spectra in a mass range from 50 to 1,300 *m*/*z* were obtained in MS and MS/MS mode. Negative electrospray ionization (ESI^−^) was used at 5 Hz with the following parameters: 500 V end plate offset, 3,000 V capillary voltage, 3 bar nebulizer pressure (N_2_), 12 L min^-1^ and 275°C of dry gas flow (N_2_) and temperature, 4 eV quadrupole ion energy, 90 *m*/*z* low mass, 7 eV collision energy in MS mode, 75 µs transfer time and 6 µs pre-pulse storage. Nitrogen served as the collision gas, fragmenting the most intense ions in Auto-MS/MS mode. Collision energies and isolation widths were ramped along with increasing *m*/*z*. A calibration solution of sodium formate was pumped through a sprayer into the ESI source before each sample to recalibrate the mass axis. Eight blanks for the larvae and fecal samples and three blanks for the control leaf discs, containing just solvent with the internal standard, were processed and analyzed in the same way, respectively.

For identification of metabolites of interest, samples with high intensities of those metabolites were measured again for specific fragmentation of these masses with a multiple reaction monitoring (MRM) method. The collision energy and isolation width were set as following: collision energy: 0.025 *m/*z* + 17.5; width: 0.005 *m/*z* + 1.5. Segments with potential metabolites were measured at 2 Hz. The samples were also measured in positive electrospray ionization (ESI^+^) mode at 2 Hz with a capillary voltage of 4,500 V, and if necessary (e.g., when no total ion chromatogram available), with a MRM-method.

A bucket table was created using MetaboScape 2021.b (Bruker). The intensity threshold was set to 750. The minimum peak length was set to seven spectra. Recursive peak picking was disabled. To sort molecular features which belong to the same metabolite into buckets, the following ion types were used [M-H]^-^, [M-H_2_O-H]^-^, [M + Cl]^-^, [M + HCOOH-H]^-^ and [2M-H]^-^. The resulting bucket table was exported to Microsoft Excel, where further filtering for possible metabolites was performed. Peak heights of the most intense metabolic features in a sample were normalized to the peak height of the internal standard, the hydrocortisone [M-HCOOH-H]^-^ ion, in the same sample. A metabolite was only kept in the data set if 1) the mean intensity of the metabolite within samples of the same treatment was at least 50 times higher than the mean intensity of the blanks for at least one group, 2) it was present in at least two out of three samples in at least one group, 3) the fold change was >2 or the metabolite was only present in the glucosinolate treatments and not in the solvent control, 4) the mean intensity was higher than the threshold of 750 counts in at least one glucosinolate treatment group and 5) metabolites were not present in the control leaf discs.

Remaining metabolites were analyzed in DataAnalysis using “SmartFormula manually” to receive sum formulas and “SmartFormula 3D” to receive ion formulas of fragments. The list of *m*/*z* and their intensities of the MS/MS spectrum were copied into MetFrag (Ruttkies et al., 2016), with PubChem as the database. In the MetFrag Scoring Term, “spectral similarity” with the MassBank of North America (MoNA; https://mona.fiehnlab.ucdavis.edu/) was enabled; the relative mass deviation for MS/MS peak match was set to 10 and the absolute mass deviation was set to 0.01. Potential structural formulas of the fragments were obtained using a combination of the results from “SmartFormula 3D”, MetFrag and information from Friedrichs et al. (2022).

For the breakdown metabolite of 1-methoxy-3-indolylmethyl glucosinolate, fragments could not be explained by MetFrag. The structure of the breakdown metabolite was constructed by comparing *m*/*z* of fragments with those of *N*-(1*H*-indol-3-ylcarbonyl) glutamic acid, which is the breakdown metabolite of indol-3-ylmethyl glucosinolate in *P. cochleariae* (Friedrichs et al., 2022). The fragments with 102, 128 and 146 *m*/*z* in ESI^−^ mode corresponded with fragments of glutamic acid in *N*-(1*H*-indol-3-ylcarbonyl) glutamic acid. The fragments with 172, 185 and 204 *m*/*z* in ESI^−^ mode and 159 and 174 *m*/*z* in ESI^+^ mode were constructed as fragments of the indole side chain of 1-methoxy-3-indolylmethyl glucosinolate using the ion formula suggested by SmartFormula 3D of Data Analysis. Since there was no information available about the possible breakdown metabolite of 1-methoxy-3-indolylmethyl glucosinolate on MetFrag or PubChem, for the remainder of this study the metabolite will be called *N*-(1-methoxy-indol-3-ylcarbonyl) glutamic acid, which is based on the naming of *N*-(1*H*-indol-3-ylcarbonyl) glutamic acid (Friedrichs et al., 2022). Further information and drawings of fragments are shown in the Supplementary Table S1.

The relative turnover of a glucosinolate (Eq. 3) was calculated as

$$Relative\;turnover=\frac{{total}_{bm}}{{total}_{bm}+{total}_{gls}}$$ (3)

with “total” being the sum of the intensity of a metabolite found in larvae and feces of the same sample and “bm” and “gls” being the breakdown metabolite and the intact glucosinolate, respectively. It must be noted that during the feeding period (ca. 24 h) feces potentially containing breakdown metabolites were not collected; the relative turnover is therefore only an approximation.

### 2.6 Statistical analyses

All statistical analyses were carried out in RStudio (version 2023.06.2 + 561) with R (version 4.2.1., R Core Team, 2022) using the packages *car* and *rstatix*. Data regarding consumption and development of the larvae were tested for normality and homoscedasticity using the Shapiro-Wilk test and Levene-test, respectively. Accordingly, data were analyzed using the *t*-test or the Mann-Whitney *U*-Test, comparing G- and G+ individuals. Due to small sample sizes, no statistical tests were performed for the data concerning the investigation of the glucosinolate metabolism.

## 3 Results

### 3.1 Larval food consumption, growth and development time

**FIGURE 2:**
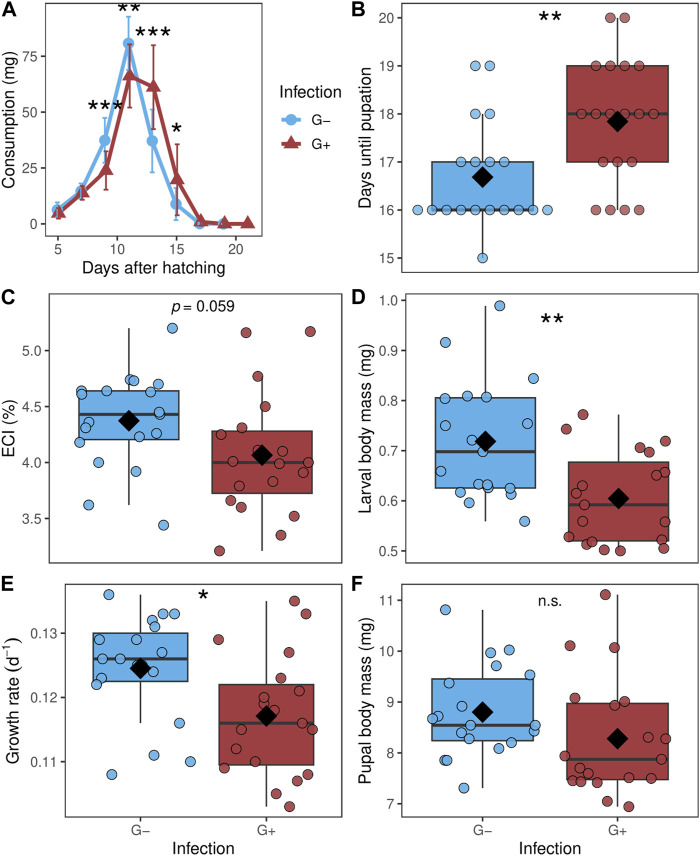
**(A)** Consumption over the larval stage from day 3 after hatching until pupation (assessed every 2 days), **(B)** days until pupation, **(C)** efficiency of conversion index (ECI), **(D)** larval body mass at day three after hatching, **(E)** growth rate and **(F)** pupal body mass of *Phaedon cochleariae* either without gregarine infection (G-) or infected with gregarines (G+). **(A)** Data is presented as means (dots) with standard deviations. **(B–F)** Data are shown as box-whisker plots with mean (diamond) and median (horizontal line), interquartile range corresponds to the size of the box, maximum and minimum values within 1.5-fold interquartile range correspond to whisker length; individual data points are shown. Asterisks indicate significant differences at different levels: *p ≤ 0.05, **p ≤ 0.01, ***p ≤ 0.001, n.s.: not significant, calculated with a **(A)** Mann-Whitney *U*-test between the two groups at one measuring event; **(B)** Mann-Whitney *U*-test **(C–F)** and *t*-test; *n* = 19 per group.

All raw data are presented in Supplementary Table S2. While the total consumption during the larval stage of *P. cochleariae* did not differ between G- and G+ individuals (data not shown), consumption measured every 2 days differed over the course of time (Figure 2A). G-larvae started to feed significantly more than G+ larvae at day 9 after larval hatching and had the highest consumption at day 11. Thereafter, on day 13, the consumption of G-larvae declined quickly while the consumption of G+ larvae remained high and was now significantly higher than that of G-individuals. Close to pupation, from day 17 onwards, both G- and G+ larvae fed only little. The developmental time until pupation was significantly shorter for G-larvae than for G+ larvae (Figure 2B). G-larvae had by trend a higher ECI than G+ larvae (Figure 2C). The larval body mass at day 3 and the growth rate were significantly higher for G-larvae than G+ larvae (Figures 2D, E). There was no significant difference for the pupal body mass of G- and G+ individuals (Figure 2F). One larva per treatment died.

### 3.2 Identified glucosinolates and corresponding metabolites in larvae and feces in dependence of gregarine infection and host plant

**FIGURE 3:**
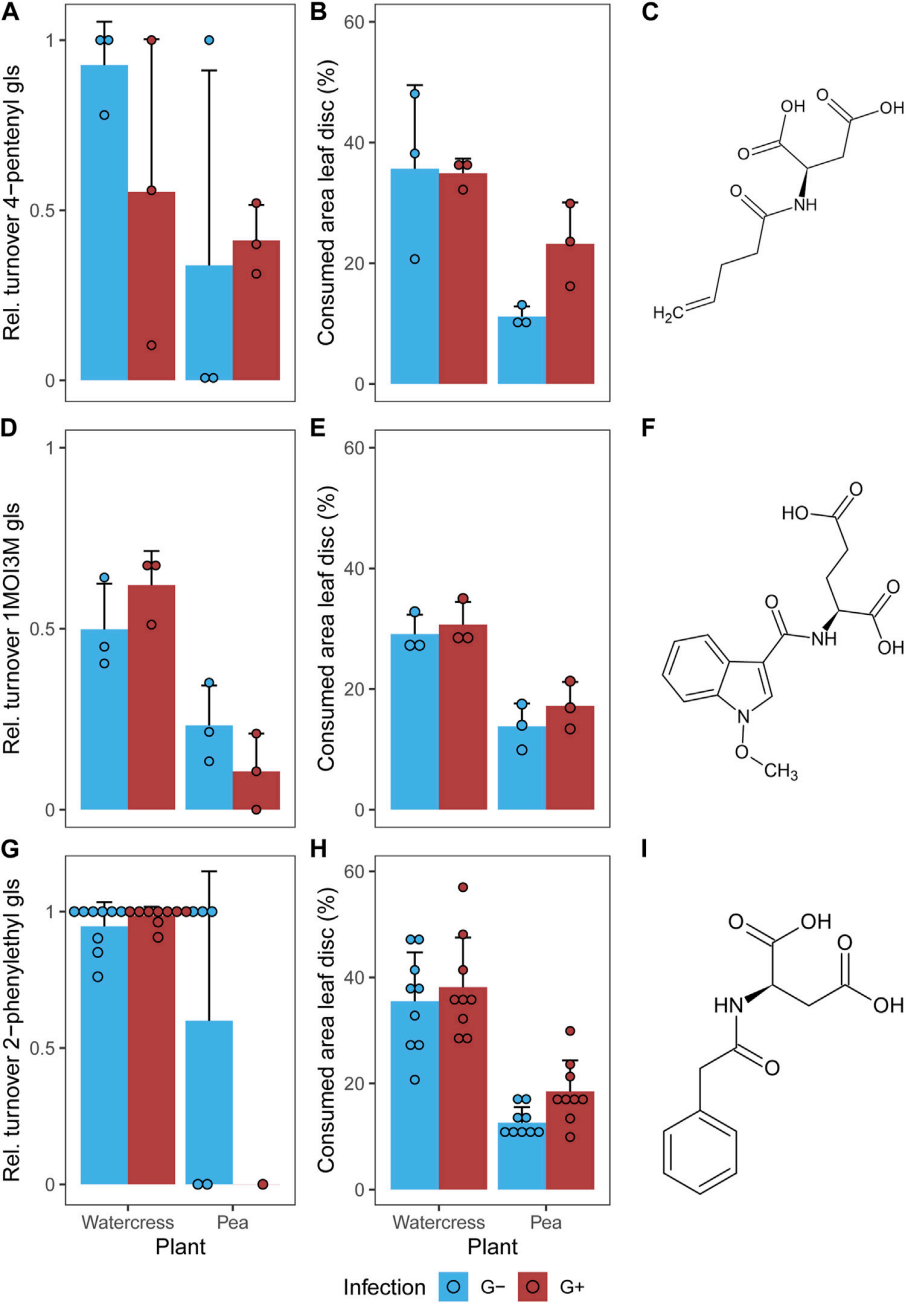
**(A,D,G)** Relative turnover of intact glucosinolates and **(B,E,H)** consumed leaf area of leaf discs **(A,B)** treated with 4-pentenyl glucosinolate (gls) or **(D,E)** 1-methoxy-3-indolylmethyl glucosinolate (1MOI3M) and **(G,H)** containing the watercress-intern 2-phenylethyl glucosinolate in larvae of *Phaedon cochleariae* either not infected (G-) or infected with gregarines (G+). Larvae were fed with glucosinolate-treated leaves of watercress (*Nasturtium officinale*) or pea (*Pisum sativum*) (*n* = 3 for **(A,B,D,E)**, *n* = 9 for **(G,H)** per gregarine infection and plant). Bar charts represent the means with standard deviations; individual data points are shown. **(C,F,I)** Structural formula of the respective breakdown metabolites are given. Please note that the larvae were allowed to feed on the leaf discs for 24 h or until ≥10% of the leaf disc had been consumed in case of feeding tests with the non-host plant pea. The feeding duration for all individuals feeding on watercress was on average 24 ± 0 h (mean ± SD), while it was 26.3 ± 6.5 h on pea.

A total of seven metabolites were identified in all samples (Table 1). Three glucosinolates were identified: the two applied 4-pentenyl glucosinolate (**1**) and 1-methoxy-3-indolylmethyl glucosinolate (**3**) as well as 2-phenylethyl glucosinolate (**6**), which is the dominant glucosinolate in watercress. As breakdown metabolites, amino acid conjugates of metabolites derived from the respective glucosinolates were found. For the aliphatic 4-pentenyl glucosinolate and the benzenic 2-phenylethyl glucosinolate, aspartic acid conjugates could be identified as breakdown metabolites, namely, *N*-(pentenoyl) aspartic acid (**2**) and *N*-(phenylacetyl) aspartic acid (**7**), respectively. For the indole 1-methoxy-3-indolylmethyl glucosinolate, two breakdown metabolites could be identified, the glutamic acid conjugate *N*-(1-methoxy-indol-3-ylcarbonyl) glutamic acid (**4**) and neoascorbigen (**5**). All breakdown metabolites of the two applied glucosinolates could be found in all samples, i.e., in individuals (and their feces) feeding on both plant species and of both gregarine treatments (Figures 3A, D). The watercress-intern glucosinolate and its breakdown metabolite could be found in both G- and G+ larvae that fed on watercress (Figure 3G). When feeding on pea, the watercress-intern glucosinolate and its breakdown metabolite could still be detected in five out of nine samples in G- but only in one out of nine samples in G+ larvae. Neoascorbigen was found in higher intensities in larval and fecal samples of larvae fed on watercress than those fed on pea (Supplementary Figure S2). Breakdown metabolites mostly had a higher intensity in feces than in larval samples, while the intact glucosinolates mostly had a higher intensity in larval samples (Supplementary Figures S1-S3). No pattern could be seen regarding the relative glucosinolate turnover in relation to the gregarine infection status (Figures 3A, D, G). The turnover of the watercress-intern 2-phenylethyl glucosinolate was by trend higher than that of both applied glucosinolates (Figures 3A, D, G).

**TABLE 1:** Glucosinolates and their respective breakdown metabolites found in larvae and feces of *Phaedon cochleariae* individuals infected or not infected with gregarines. Larvae were fed with treated watercress (*Nasturtium officinale*) or pea (*Pisum sativum*) leaf discs. The molecular formulas, average retention times (RT; in ESI^−^ and ESI^+^ mode) as well as dominant ion types, *m*/*z* and ion formulas for ESI^+^ and ESI^−^ mode are given. If a metabolite could not be detected in any sample, it is given as “not found”. For additional information about e.g., fragmentation pattern see Supplementary Table S1.

ID	Metabolite	Molecular formula	RT [min]	ESI^-^			ESI^+^		
				Ion type	*m*/*z*	Ion formula	Ion type	*m*/*z*	Ion formula
**1**	4-pentenyl glucosinolate	C_12_H_21_NO_9_S_2_	4.40	[M-H]^-^	386.0589	[C_12_H_20_NO_9_S_2_]^-^		not found	
**2**	*N*-(4-pentenoyl) aspartic acid	C_9_H_13_NO_5_	3.30	[M-H]^-^	214.0720	[C_9_H_12_NO_5_]^-^		not found	
**3**	1-methoxy-3-indolylmethyl glucosinolate	C_17_H_22_N_2_O_10_S_2_	10.70	[M-H]^-^	477.0648	[C_17_H_21_N_2_O_10_S_2_]^-^		not found	
**4**	*N*-(1-methoxy-indol-3-ylcarbonyl) glutamic acid	C_15_H_16_N_2_O_6_	13.90	[M-H]^-^	319.0936	[C_15_H_15_N_2_O_6_]^-^	[M+H]^+^	321.1086	[C_15_H_17_N_2_O_6_]^+^
**5**	neoascorbigen	C_16_H_17_NO_7_	14.30	[M-H]^-^	334.0934	[C_16_H_16_NO_7_]^-^	[M+H]^+^	336.1069	[C_16_H_18_NO_7_]^+^
**6**	2-phenylethyl glucosinolate	C_15_H_21_NO_9_S_2_	7.40	[M-H]^-^	422.0583	[C_15_H_20_NO_9_S_2_]^-^		not found	
**7**	*N*-(phenylacetyl) aspartic acid	C_12_H_13_NO_5_	6.65	[M-H]^-^	250.0721	[C_12_H_12_NO_5_]^-^	[M+H]^+^	252.0866	[C_12_H_14_NO_5_]^+^

## 4 Discussion

### 4.1 Larval food consumption, growth and development

The food consumption over the whole larval stage did not differ between G- and G+ larvae of *P. cochleariae*, but the consumption over the course of time differed. G-larvae started and stopped eating large amounts earlier and showed a faster growth rate than G+ larvae. Likewise, the ECI was at least by trend a bit higher in G-larvae, indicating a slightly more efficient food to energy conversion than in G+ larvae. Thus, the hypothesis that G+ larvae have to take up more food but gain less body mass than G-larvae can only be partly supported. In a previous study with *P. cochleariae*, the food consumption of 9 days old larvae measured over 24 h was not affected by gregarine infection (Wolz et al., 2022b). Likewise, in *Forficula auricularia* (Dermaptera: Forficulidae), the food consumption, only assessed for 1 h, was not influenced by the presence and number of gregarines (Arcila and Meunier, 2020). The present study highlights that differences in food consumption in dependence of gregarine infection may only become evident if measured for a longer duration. The tendency for a lower ECI in G+ larvae of *P. cochleariae* may be explained by a potential blockage of the host gut or damage of the midgut epithelium by trophozoites, as found in the damselfly *Pyrrhosoma nymphula* (Coenagrionoidea: Coenagrionidae) (Åbro, 1971). Moreover, G+ larvae may lose nutrients to the trophozoites (Valigurová, 2012; Valigurová and Florent, 2021), which can therefore no longer be used for their own growth.

Similarly to the lower growth rate and larval body mass in G+ larvae, a reduced larval size could be found in *T. castaneum* under gregarine infection (Gigliolli et al., 2016). Contrasting to this, in *Gryllus veletis* and *G. pennsylvanicus* (Orthoptera: Gryllidae) no significant differences in body mass change were found between infected and uninfected individuals. However, the two *Gryllus* species were weighed over a few weeks as adults, in which body mass changes are probably less pronounced than during juvenile stages. Although the larval growth rates differed between G- and G+ individuals of *P. cochleariae*, the pupal body mass (present study) and the adult body mass (Wolz et al., 2022a; Wolz et al., 2022b) were not affected by the gregarine infection. Thus, potential impacts on nutrition by gregarines during the larval stage did not have long-lasting effects at least on the body mass. This compensation in G+ larvae may have occurred because even though it took them longer, when reaching pupation G+ larvae had consumed the same amount as G-larvae. The difference in the ECI was apparently not large enough to lead to differences in pupal body mass.

Gregarine infection prolonged the developmental time until pupation, supporting our hypothesis. This delayed development may have resulted from the delayed increase in consumption over time. Previous studies on *P. cochleariae* found likewise a delayed development under gregarine infection (Wolz et al., 2022b) or also no differences in development time until adulthood (Wolz et al., 2022a). Different generations of *P. cochleariae* were used in the different experiments and therefore infection intensities may have differed among these. Furthermore, the effect of gregarines on developmental time appears to be species-specific. Similar to the present study, a longer developmental time under gregarine infection was found in *T. castaneum* (Gigliolli et al., 2016), *G. veletis* and *G. pennsylvanicus* (Zuk, 1987). In contrast to those species, an acceleration of development was shown in *Ctenocephalides felis* (Siphonaptera: Pulicidae) when infected with gregarines (Alarcón et al., 2017). These differences may be either due to different sensitivities of the host species or different costs or benefits imposed by the gregarine species.

It should be noted that the longer development of G+ compared to G-larvae in the present study could also be due to the lower quality of reinfection leaves provided to G+ larvae for the first 2 days after hatching. On those leaves, adult beetles had already fed upon, while the control leaves offered to G-larvae during that time period were only mechanically damaged. Since G-larvae had a higher larval body mass already at day 3 after hatching, food quality may indeed have played an important role. It is well known that the development of *P. cochleariae* is strongly influenced by plant quality (Tremmel and Müller, 2013). Thus, in future studies the role of food quality *versus* gregarine infection on the development of the host should be disentangled. To better understand the effects of gregarine infection levels over multiple generations, short- and long-term effects of a gregarine infection on life history traits of *P. cochleariae* could be investigated.

### 4.2 Identified glucosinolates and corresponding metabolites in larvae and feces in dependence of gregarine infection and host plant

The metabolism of structural different glucosinolates by *P. cochleariae* resulted in amino acid conjugates, which were found in the larvae and their feces. Intermediate breakdown metabolites of the aliphatic 4-pentenyl glucosinolate and the benzenic 2-phenylethyl glucosinolate were both conjugated with aspartic acid, contrasting our expectation of a conjugation of those classes with amino acids different from each other. However, the metabolite derived from indole 1-methoxy-3-indolylmethyl glucosinolate was conjugated with glutamic acid. A conjugation of intermediate breakdown metabolites, most likely ITCs, of benzenic glucosinolates with aspartic acid and of that of another indole glucosinolate with glutamic acid by *P. cochleariae* has been described before (Friedrichs et al., 2020; Friedrichs et al., 2022), while the metabolism for aliphatic glucosinolates had been unknown.

Neoascorbigen was another metabolite found in larvae fed with leaf discs treated with 1-methoxy-3-indolylmethyl glucosinolate in the present study. This is consistent with the results of a previous study (Friedrichs et al., 2022), in which ascorbigen was found as a metabolite in larvae fed with indol-3-ylmethyl glucosinolate-treated leaves and indicates that *P. cochleariae* conjugates breakdown products of indole glucosinolates also with ascorbate. However, ascorbigen can also form spontaneously from the unstable ITCs of indole glucosinolates (Blažević et al., 2020). Ascorbigen was also found in leaves of watercress, but only in one pea leaf in a very low concentration in Friedrichs et al. (2022). This may explain why neoascorbigen was mainly found in larvae and feces of larvae that were fed with watercress but not in those fed with pea (Supplementary Figure S2), but note that in the present study neoascorbigen was not found in any control leaf samples. In the gut of the larvae, the watercress-intern myrosinases may have hydrolyzed 1-methoxy-3-indolylmethyl glucosinolate into the respective ITC, which could have spontaneously turned into neoascorbigen because of the unstable character of the ITC. Nevertheless, some neoascorbigen was also found in samples of larvae fed with pea, in which no myrosinases are present and ITCs should not be formed. But note that the watercress-intern glucosinolate and its breakdown metabolite were also found in some samples of larvae fed with pea (Figure 3 and Supplementary Figure S3), indicating that gut contents may not have been completely emptied during molting. In line with this, recent findings on two other beetle species indicate that even during metamorphosis the adult gut forms a new layer around the larval gut and its content (Vommaro et al., 2024). However, it is also noteworthy that after feeding on watercress, no myrosinase activity was found in freshly molted and starved larvae in comparison to larvae only starved, indicating that plant residues are likely excreted before/during molting (Friedrichs et al., 2020). G+ larvae may also empty their guts more completely than G-larvae, hinting at an influence of gregarines on the shedding of gut contents during molting. Across all samples investigated here, breakdown metabolites were mostly found in the feces, while the intact precursor glucosinolates were mostly found in the larval samples (Supplementary Figures S1-S3), indicating that most of the glucosinolates are metabolized and then readily excreted. The larvae seemed to be better at metabolizing the watercress-intern glucosinolate compared to both of the applied glucosinolates (Figures 3A, D, G). This may be explained by the fact that *P. cochleariae* was collected in the wild from watercress and also kept on this plant species in the lab for several generations; therefore the individuals may be more accustomed to the plant’s dominant glucosinolate. Host plant switches (and therefore switches in glucosinolate composition) have been shown to influence gene expression related to metabolism and digestion in *P. cochleariae* (Müller et al., 2017). This may also explain the somewhat higher variation in glucosinolate turnover in some samples of the applied glucosinolates. The high variation in turnover may, furthermore, indicate a high variation in individual capacity of larvae to metabolize glucosinolates they are usually not confronted with. Also, the applied glucosinolates may not have been distributed completely evenly on the leaf disc, which could explain some variation in turnover rates, especially when the feeding amount was small. Our method used for filtering metabolites of interest focused on those metabolites highly abundant in the larvae and fecal samples that could not be found in the control leaves, to avoid picking plant metabolites. It can therefore not be ruled out that we missed metabolites with only minor changes in larvae/feces or metabolites which are only detectable with platforms other than the one used here. In future studies, a combination of glucosinolates could also be applied on the leaf discs to account for potential effects of mixtures of glucosinolates and competition for amino acids for conjugation to their breakdown metabolites.

The present study does not support an essential role of gregarines acting as mutualists in the metabolism of glucosinolates in *P. cochleariae*. Glucosinolates first need to be hydrolyzed to ITCs/CNs to detoxify those by conjugation of amino acids to further breakdown metabolites (Friedrichs et al., 2020). This amino acid conjugation to intermediate breakdown metabolites of glucosinolates also happens without plant intern myrosinases, therefore suggesting that a gut symbiont other than gregarines may be taking over this function. In *P. cochleariae*, various genes associated with bacteria and yeasts could be found (Müller et al., 2017). Gut symbionts can often contribute to the host’s digestion or detoxification of specialized plant metabolites, as they are directly exposed to the ingested plant material (Itoh et al., 2018). However, for symbionts that are only horizontally transmitted, as is the case for many insect gut symbionts (Kucuk, 2020), a transfer to conspecific hosts may be too unreliable for important detoxification functions (Hansen and Moran, 2014). Bacterial lineages often show dynamic genomes needed for quick adaptations such as changes of host plants (Hansen and Moran, 2014). This may be the reason why bacteria were often found to either have direct effects on their host’s detoxification or enhance the activity of enzymes or gene expression involved in food digestion or detoxification of specialized metabolites (Itoh et al., 2018; Zhao et al., 2022). Some fungi are also able to detoxify plant metabolites (Wadke et al., 2016). For example, *Sclerotinia sclerotiorum* is able to degrade ITCs through hydrolysis and the mercapturic acid conjugation pathway to circumvent their harmful effects (Chen et al., 2020). Different bacteria and fungi were found in the gut microbiome of *Altica fragariae* and *A. viridicyanae* (Coleoptera: Chrysomelidae) that are known to be able to degrade specialized plant metabolites, such as triterpenoids, flavonoids and glycosides (Wei et al., 2022). Further studies are needed to find out whether and which microbiota may fulfill the function of hydrolyzing glucosinolates even when plant myrosinases are absent in *P. cochleariae*.

## 5 Conclusion

In this study, gregarines were found to have negative effects on the development and growth rate of *P. cochleariae* larvae. They did not show an effect on the ability of *P. cochleariae* larvae to metabolize glucosinolates in the presence or absence of plant-derived myrosinases. Therefore, concerning the parameters examined in this study, gregarines would rather be placed on the parasitic side of the parasitism-mutualism spectrum. Moreover, a possible metabolism of an aliphatic glucosinolate could be revealed in *P. cochleariae*. The amino acids aspartic acid and glutamic acid are conjugated to breakdown products of glucosinolates by *P. cochleariae* to circumvent potential toxic effects.

## Data Availability

Metabolomics data have been deposited to the EMBL-EBI MetaboLights database (DOI: 10.1093/nar/gkad1045, PMID:37971328) with the identifier MTBLS9816. The complete dataset can be accessed here https://www.ebi.ac.uk/metabolights/MTBLS9816.
