# Supplementary material for: Gregarines impact consumption and development but not glucosinolate metabolism in the mustard leaf beetle
Source: Front Physiol. 2024 May 1;15:1394576. doi: 10.3389/fphys.2024.1394576 (PMC11094291; doi:10.3389/fphys.2024.1394576)
Supplement: Supplementary file 2 [file DataSheet1.PDF]

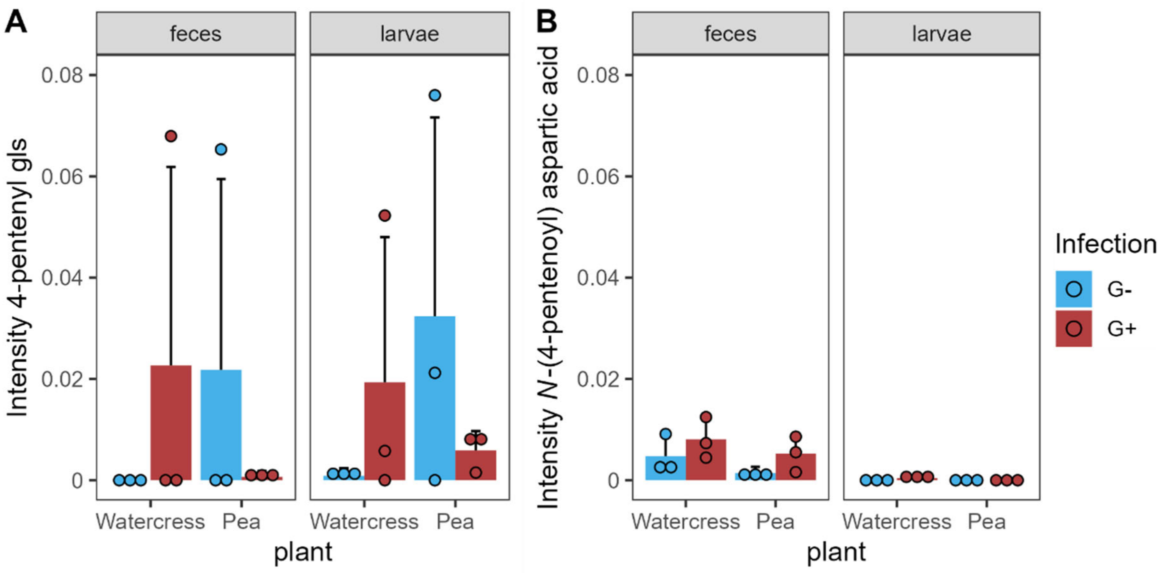

Fig. S1: Normalized peak intensities of **(A)** the intact 4-pentenyl glucosinolate (gls) and **(B)** its breakdown metabolite *N*-(4-pentenoyl) aspartic acid in feces and larval samples of *Phaedon cochleariae* either not infected (G-) or infected with gregarines (G+). Larvae were fed with either glucosinolate-treated watercress (*Nasturtium officinale*) or pea (*Pisum sativum*) leaves (*n* = 3 per gregarine infection and plant). The bar charts represent the means with standard deviations; individual data points are shown.

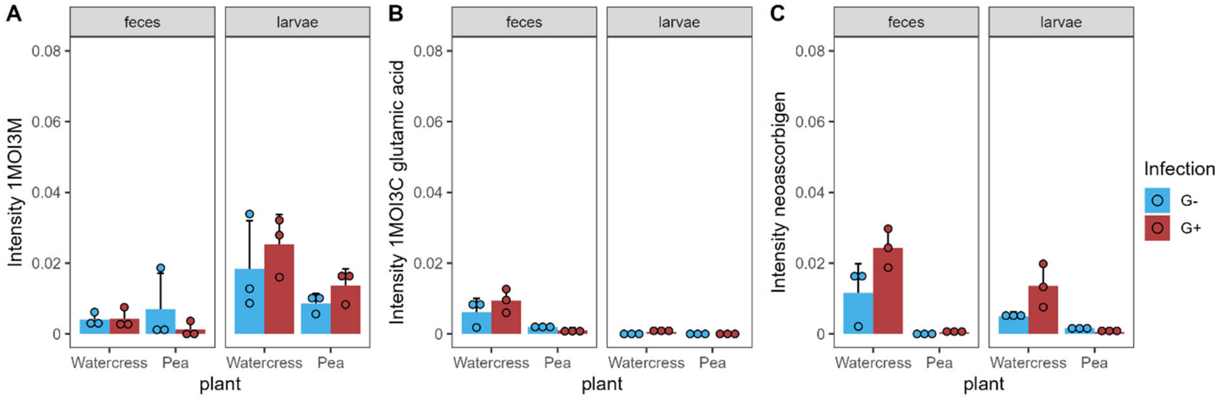

Fig. S2: Normalized peak intensities of **(A)** the intact 1-methoxy-3-indolylmethyl glucosinolate (1MOI3M) and its breakdown metabolites **(B)** *N*-(1-methoxy-indol-3ylcabonyl) glutamic acid (1MOI3C) and **(C)** neoascorbigen in feces and larval samples of *Phaedon cochleariae* either not infected (G-) or infected with gregarines (G+). Larvae were fed with either glucosinolate-treated watercress (*Nasturtium officinale*) or pea (*Pisum sativum*) leaves (*n* = 3 per gregarine infection and plant). The bar charts represent the means with standard deviations; individual data points are shown.

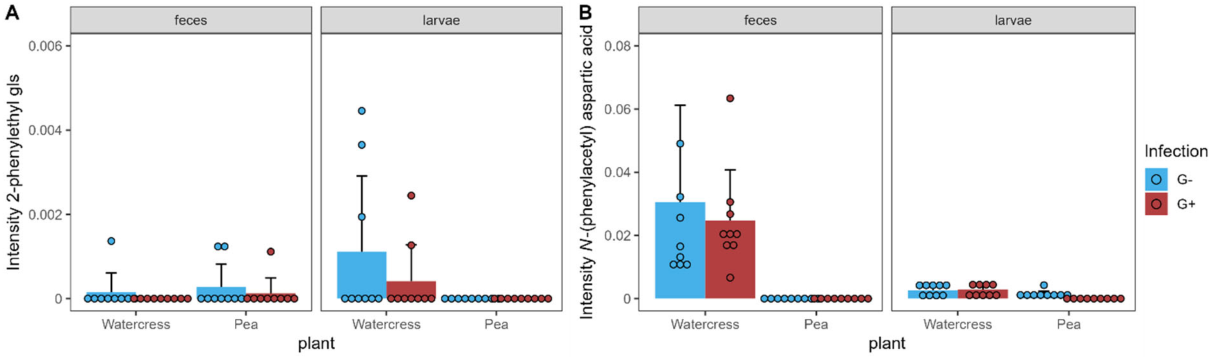

Fig. S3: Normalized peak intensities of **(A)** the intact watercress-intern 2-phenylethyl glucosinolate (gls) and **(B)** its breakdown metabolite *N*-(phenylacetyl) aspartic acid in feces and larval samples of *Phaedon cochleariae* either not infected (G-) or infected with gregarines (G+). Larvae were fed with either glucosinolate-treated watercress (*Nasturtium officinale*) or pea (*Pisum sativum*) leaves (*n* = 9 per gregarine infection and plant). The bar charts represent the means with standard deviations; individual data points are shown.
